# Clinicopathological significance of homeoprotein Six1 in hepatocellular carcinoma

**DOI:** 10.1038/sj.bjc.6603399

**Published:** 2006-09-26

**Authors:** K T Ng, K Man, C K Sun, T K Lee, R T Poon, C-M Lo, S-T Fan

**Affiliations:** 1 Centre for the Study of Liver Disease and Departments of Surgery, The University of Hong Kong, Queen Mary Hospital, L9-55, Faculty of Medicine Building, 21 Sassoon Road, Pokfulam, Hong Kong, China

**Keywords:** hepatocellular carcinoma (HCC), Six1, hepatectomy, pathologic tumour-node-metastasis (pTNM) stage, venous infiltration, survival

## Abstract

Tumour recurrence and metastases of hepatocellular carcinoma (HCC) after hepatectomy are the major obstacles of long-term survival. The present study investigated the clinicopathological significance of a possible metastasis regulator Six1 in HCC patients who were undergone hepatectomy. Seventy-two pairs of RNA and 103 pairs of protein from tumour and adjacent nontumour liver tissues of HCC patients were examined. About 85 and 60% of HCC tumour tissues were found to overexpress Six1 mRNA and protein, respectively, compared with nontumour liver tissues. No Six1 protein was detected in HCC nontumour liver tissues and normal liver tissues. Increased Six1 protein expression in HCC patients was significantly correlated with pathologic tumour-node-metastasis (pTNM) stage (*P*=0.002), venous infiltration (*P*=0.004) and poor overall survival (*P*=0.0423). We concluded that Six1 is frequently overexpressed in HCC patients and elevated Six1 protein in HCC patients may be an indication of advanced stage and poor overall survival after hepatectomy.

Hepatocellular carcinoma (HCC) is one of the most malignant cancers in the world with estimated 350 000 new cases and nearly one million deaths annually (Schafer and Sorrell, 1999; Okuda, 2000). Acceleration of hepatitis B virus and hepatitis C virus infection resulted in increase of HCC incidence both in Europe and the US (El-Serag and Mason, 1999; Bosch *et al*, 2004). Surgical treatments in terms of hepatic resection and orthotopic liver transplantation are the potentially curative treatments for HCC, but the long-term disease-free survival remains unsatisfactory (Lo and Fan 2004; Poon and Fan 2004). Tumour recurrence and metastases are the major causes of death in HCC patients after surgical treatments (Llovet *et al*, 2005), indicating the necessity of developing new therapeutic strategies targeting at tumour recurrence and metastases in HCC. So far, the molecular mechanism governing these processes in HCC remains unclear; hence continuous identification and characterisation of novel metastasis-associated genes are indispensable.

Six1 belongs to a subfamily of the Six class of homeodomain-containing transcription factors, which share a lysine within the DNA-binding helix of the homeodomain (Oliver *et al*, 1995). The functions of vertebrate Six1 are involved in diverse organ developments of the brain, ear, eye, muscle and kidney (Relaix and Buckingham, 1999; Laclef *et al*, 2003; Xu *et al*, 2003; Zheng *et al*, 2003; Ozaki *et al*, 2004). Overexpression of Six1 occurs in a large percentage of primary breast cancer and strongly correlates with metastatic breast lesions (Ford *et al*, 1998). Six1 elevated in breast cancer promotes tumour progression through direct activation of Cyclin A1 (Coletta *et al*, 2004). Moreover, Six1 plays a substantial role in regulating the metastatic ability of rhabdomyosarcoma (RMS) (Yu *et al*, 2004). The above evidences indicate that Six1 may be a critical regulator of metastases in different cancers. Up to now, there has been no research reporting its roles in HCC. In this study, we provided the first evidence of Six1 expression in HCC patients and cell lines aiming to investigate its clinicopathological significance in HCC.

## MATERIALS AND METHODS

### Cell lines

Nonmetastatic human HCC cell lines Hep3B, Huh7 and PLC were purchased from the American Type Culture Collection (Manassas, VA, USA), and metastatic human HCC cell lines MHCC97L and MHCC97H were obtained from the Liver Cancer Institute and Zhongshan Hospital of Fudan University, Shanghai, the People's of Republic of China (Li *et al*, 2001). All cells were cultured in DMEM high glucose medium with 10% FBS (Gibco, Grand Island, NY, USA) and 1% penicilium and streptomycin at humidified 37°C incubator supplied with 5% CO_2_.

### Clinical samples

One hundred and three HCC patients undergone liver resection between March 1999 and June 2004 were recruited from Department of Surgery, Queen Marry Hospital, the University of Hong Kong, contributing to 103 pairs of protein extracts and 72 pairs of RNA extracts from tumour tissues and adjacent nontumour tissues. There were 87 men and 16 women. The age of the patients ranged from 33–75 years, with a median age of 55 years. Eighty-six HCC patients (83.5%) were positive for hepatitis B surface antigen, whereas only three (2.9%) were positive for hepatitis C virus antibody. Twenty normal liver tissues were recruited from living donors at the same hospital. All donors were examined to be free of liver diseases and hepatitis B infection. The follow-up duration for the HCC patients ranged from July 1999 to November 2005. The study was approved by the Ethics Committee of the University of Hong Kong.

### Reverse transcription–polymerase chain reaction

Total RNA from liver tissues and cell lines were extracted by TRIZol regent according to the manufacturer's instruction (Invitrogen, Carlsbad, CA, USA). Each complementary DNA was synthesised from 1 *μ*g of RNA extract using the High capacity cDNA Kit (Applied Biosystems, Foster City, CA, USA) under the condition of 25°C for 5 min following by 37°C for 2 h. For clinical samples, polymerase chain reaction (PCR) reaction for Six1 was performed using the Taq PCR Kit (Promega, Madison, WI, USA) under the following PCR cycles: 95°C for 5 min, 40 cycles at 95°C for 1 min, 57°C for 1 min and 72°C for 1 min. For internal control 18S, 30 PCR cycles were used. For cell line samples, 30-cycle multiplex PCR reaction was performed by combining Six1 and 18S primers. Polymerase chain reaction products were visualised by 2% agarose gel electrophoresis stained with ethidium bromide. Primers sets used were as follows: for Six1, sense 5′ AAG GAG AAG TCG AGG GGT GT-3′, antisense 5′-TGC TTG TTG GAG GAG GAG TT-3′; for 18S ribosomal RNA, sense 5′-CTC TTA GCT GAG TGT CCC GC-3′, antisense 5′-CTG ATC GTC TTC GAA CCT CC-3′.

### Western blot

Proteins from clinical specimens and HCC cell lines were prepared by using Urea buffer (8 M Urea, pH 8.0). The amount of protein lysates used for Western blot analysis was 100 mg and 50 mg for clinical HCC samples and HCC cell lines, respectively. Protein extracts were separated by 12% SDS–PAGE and transferred to PDMF membrane (Millipore, Billerica, MA, USA) according to the standard protocol. After blocking with 5% nonfat milk at room temperature for 1 h, antibody with proper dilution was hybridised with the membrane at 4°C for overnight. The membrane was washed three times with TBS/T each for 10 min and incubated with secondary antibody at room temperature for 1 h. Protein signal was detected by ECL Plus system (Amersham Biosciences, Piscataway, NJ, USA). Antibodies Six1 and *β*-actin were purchased from Santa Cruz Biotechnology (Santa Cruz, CA, USA).

### Statistical analysis

Statistical analysis was carried out using SPSS 11.5. for Windows (SPSS Inc., Chicago, IL, USA). The association of Six1 protein expression and clinicopathological parameters including sex, age, tumour-node-metastasis (pTNM) staging, venous infiltration, encapsulation, tumour size, alpha-fetoprotein level and hepatitis B surface antigen was analysed by *χ*^2^-test. The prognostic value of Six1 protein for predicting the overall survival of HCC patients after hepatic resection was calculated by Kaplan–Meier analysis with the log-rank test. Cox proportional hazard regression model was performed with univariable and multivariable analyses to test factors that were significantly associated with the overall survival of the HCC patients. Logistic regression analysis was also performed to compare those factors for predicting 1- and 5-year overall survival of the HCC patients. *P* value <0.05 was considered to be statistically significant.

## RESULTS

### Six1 expression in HCC cell lines

**Figure 1:**
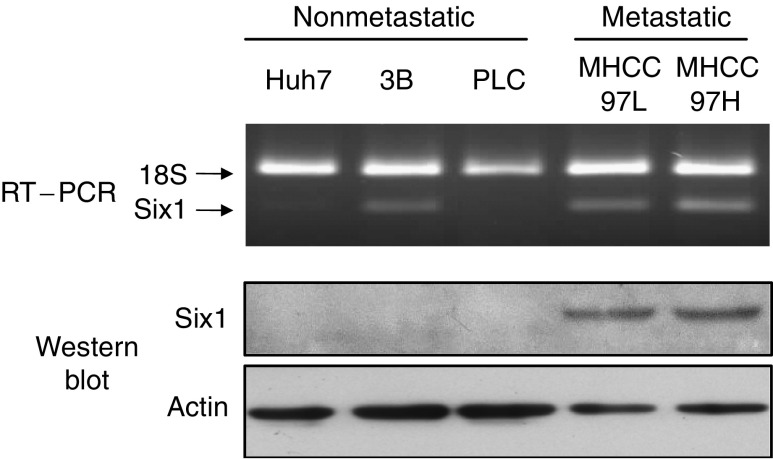
Expression pattern of Six1 gene among nonmetastatic (Hep3B, Huh7 and PLC) and metastatic (MHCC97L and MHCC97H) HCC cells. Six1 mRNA was detected by RT–PCR and Six1 protein was detected by Western blot analysis. Ribosomal RNA 18S and *β*-actin were internal control for RT–PCR and Western blot, respectively.

Three nonmetastatic HCC cell lines (Hep3B, Huh7 and PLC) and two metastatic HCC cell lines (MHCC97L and MHCC97H) were checked for Six1 gene expression in terms of mRNA and protein levels (Figure 1). Using multiplex RT–PCR analysis, Six1 mRNA was detected with a relatively higher level in Hep3B, MHCC97L and MHCC97H comparing with the mRNA level in Huh7 and PLC. The metastatic HCC cell line MHCC97H that is with the highest metastatic potential expressed the highest level of Six1 mRNA. Western blot analysis showed that Six1 protein was only expressed in the metastatic cells and expressed the highest level in MHCC97H.

### Six1 expression in HCC patients and normal donors

**Figure 2:**
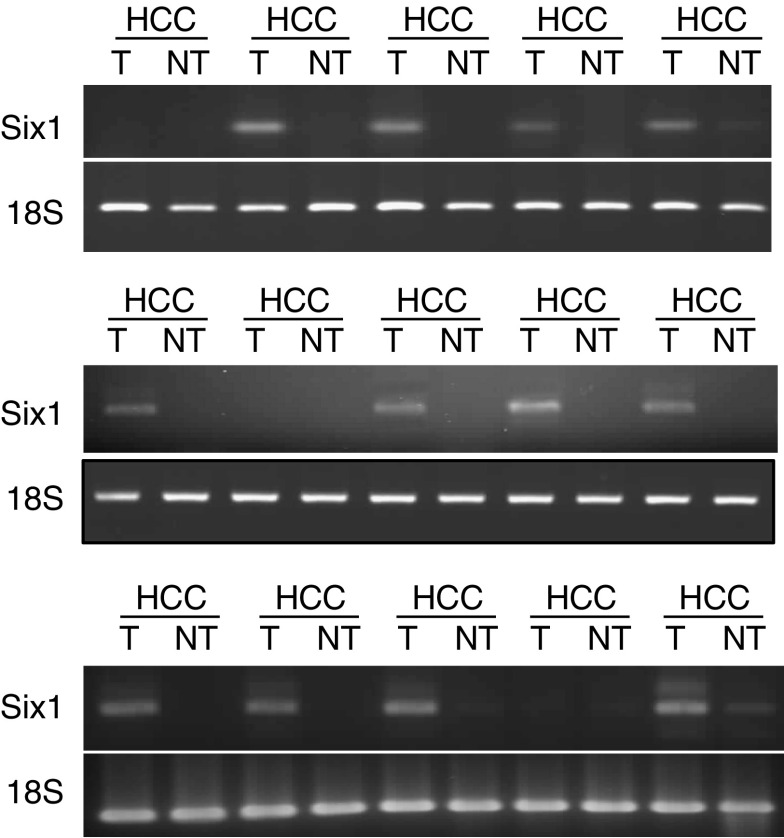
mRNA expression pattern of Six1 gene in HCC patients. RT–PCR amplification was performed to exam the Six1 mRNA level in HCC tumour tissues (T) and paired nontumour tissues (NT). Ribosomal RNA 18S was used as internal control.

**Figure 3:**
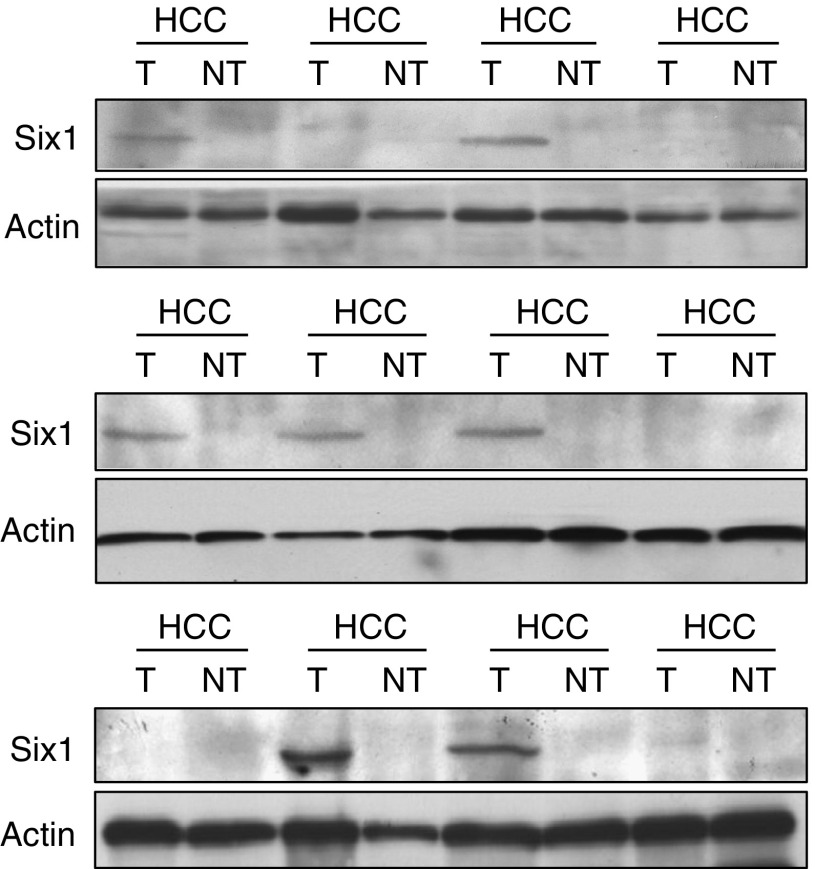
Six1 protein expression pattern in HCC patients. Western blot analysis was performed to exam the Six1 protein in HCC tumour tissues (T) and paired nontumour tissues (NT). *β*-actin was used as internal control.

Seventy-two pairs of tumour and nontumour liver tissues were subjected for detection of Six1 mRNA by RT–PCR (Figure 2). A single-pair primer with 40-PCR-cycle amplification was employed because Six1 mRNA in clinical samples could not be detected by using multiplex PCR condition. Overexpression of Six1 mRNA was found in about 85% (61 of 72) of tumour tissues compared with nontumour tissues (Table 1). Most of the nontumour liver tissues (91.7%) showed no Six1 mRNA expression. A few nontumour tissues (six of 72) expressed Six1 mRNA but all the expression levels were lower than those in the matched tumour tissues. Western blot analysis showed that near 60% (61 of 103) of tumour tissues expressed Six1 protein while no nontumour tissue expressed Six1 protein (Figure 3, Table 1). Using *χ*^2^-test with Fisher's exact test, Six1 mRNA overexpression in HCC patients was significantly correlated with their Six1 protein overexpression (*P*=0.000, *r*=0.438). All tumour tissues with positive Six1 protein expression could match with their positive mRNA expression except one case.

**Figure 4:**
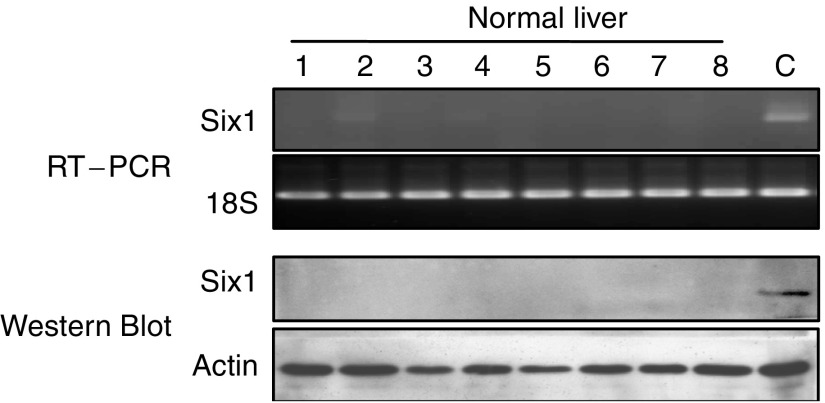
RT–PCR amplification and Western blot analysis of Six1 gene in normal liver tissues. 1–8, liver tissues from different normal donors; C, positive control.

Reverse transcription–polymerase chain reaction and Western blot were also used to analyse the Six1 mRNA and protein expression patterns in normal liver tissues (Figure 4, Table 1). Most of the normal liver tissues showed no Six1 mRNA expression (90%), whereas only a few of them (two of 20) showed weak positive signal. Moreover, no Six1 protein was expressed in normal liver tissues.

### Six1 protein expression correlated with advanced tumour stage

Six1 protein expression pattern in HCC tumour tissues was compared with their clinicopathological features (Table 2). Overexpression of Six1 protein was significantly correlated with pTNM stage (*P*=0.002, *r*=0.312) and venous infiltration (*P*=0.004, *r*=0.282). Six1 protein expression was detected in 71% (45 of 63) of advanced stage of HCC patients and in 71% (42 of 59) of HCC patients with venous infiltration. No significant association was found between Six1 protein and sex, age, cirrhosis, encapsulation, tumour size, alpha-fetoprotein level or hepatitis B surface antigen.

### Six1 protein expression correlated with poor survival

**Figure 5:**
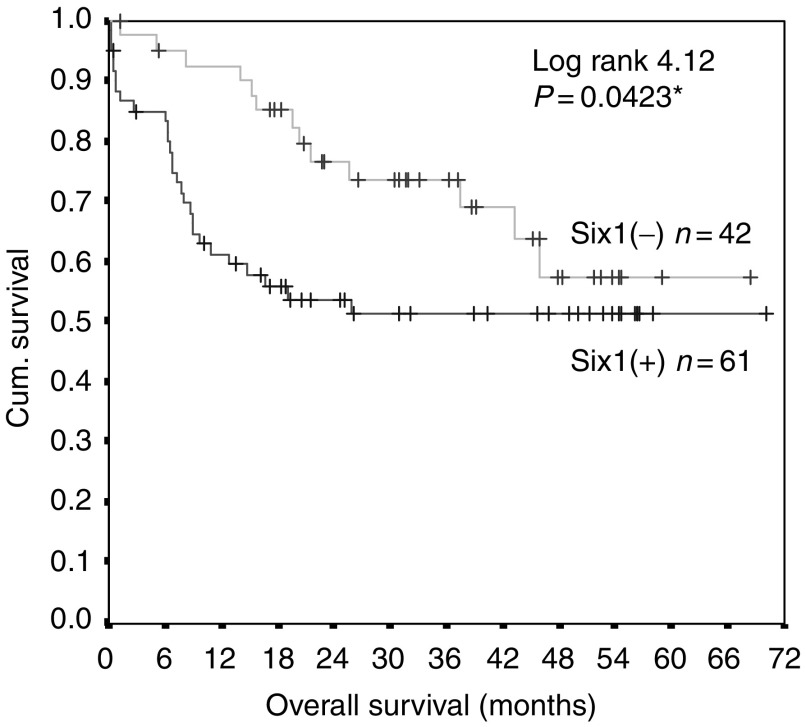
Kaplan–Meier overall survival curve of HCC patients in correlation with Six1 protein expression.

Kaplan–Meier analysis was employed to analyse the overall survival rate of HCC patients in correspondence to the Six1 protein expression pattern. The result showed that HCC patients who overexpressed Six1 protein were significantly associated with poor overall survival (log rank=4.12, *P*=0.0423, Figure 5). Kaplan–Meier analysis of pTNM staging, venous infiltration and alpha-fetoprotein level also revealed to be significantly associated with the overall survival of HCC patients (data not shown). To further examine which factors were the independent predictors, univariable and multivariable Cox proportional hazard regression analyses of these factors corresponding to the overall survival of HCC patients were performed. Univariable Cox proportional hazard regression analysis showed that Six1 protein (HR=1.956, 95% CI 1.011–3.784, *P*=0.046) and other factors were significantly associated with the overall survival of HCC patients after hepatic resection (Table 3). However, multivariable analysis indicated that pTNM staging was the only independent factor for predicting the overall survival of HCC patients (HR=7.698, 95% CI 1.891–31.33, *P*=0.004, Table 3). Logistic regression analysis of these factors associated with the 1- and 5-year overall survival showed that Six1 protein was the most influential factor for predicting the 1-year overall survival (OR=5.405, 95% CI 1.427–20.474, *P*=0.013) while pTNM staging was the most important factor for predicting the 5-year overall survival (OR=12.152, 95% CI 1.652–89.378, *P*=0.004, Table 4).

## DISCUSSION

Homeobox transcription factor Six1, which is located at 14q23 of the chromosome, is involved in the development of many organs including the brain, ear, eye, muscle and kidney (Relaix and Buckingham, 1999; Laclef *et al*, 2003; Xu *et al*, 2003; Zheng *et al*, 2003; Ozaki *et al*, 2004). Alteration of Six1 expression takes place in human breast and Wilms' cancer as well as RMS, indicating its possible contribution in the tumorigenicity of different cancers (Ford *et al*, 1998; Li *et al*, 2002; Yu *et al*, 2004). Six1 is overexpressed in 44% of primary tumours and 90% of metastatic tumours in breast cancer (Ford *et al*, 1998). Moreover, elevation of Six1 in human RMS patients is significantly associated with a later tumour stage (Yu *et al*, 2004). To investigate whether Six1 is also deregulated in HCC patients, both mRNA and protein levels of Six1 were examined in this study. Agreed with other studies, we found that overexpression of Six1 frequently occurred in tumour tissues of human HCC patients in terms of about 85% in mRNA level and 60% in protein level (Table 1). Although overexpression of Six1 mRNA in tumour tissues of HCC patients was significantly correlated with their positive Six1 protein expression, the percentage of Six1 protein overexpression in tumour tissues was lower than the percentage of Six1 mRNA overexpression. This phenomenon was also observed in HCC cell lines, in which Hep3B expressed Six1 mRNA but not Six1 protein (Figure 1). Furthermore, a small portion of HCC nontumour liver tissues (8.3%) and normal liver tissues (10%) showed positive Six1 mRNA expression rather than protein expression (Table 1). All these data suggested that the post-transcriptional regulation of Six1 among HCCs may be different and Six1 protein level rather than mRNA level is more likely to reflect its real expression status. In addition, expression of Six1 protein in liver is probably to be tumour-specific indicated by the evidence that no Six1 protein was expressed in both normal liver tissues and HCC nontumour liver tissues (Table 1). The tumour-specific characteristic of Six1 in HCC may thus provide a useful implication for therapeutic application of Six1 in HCC by suppression strategy.

Overexpression of Six1 is potentially related to metastatic status of breast cancer and RMS (Ford *et al*, 1998; Yu *et al*, 2004). MHCC97H and MHCC97L are human HCC cell lines with the same genetic background but with different local and distant metastatic potentials (Li *et al*, 2001). Orthotopic implantation of MHCC97H cell into nude mice results in 100% of lung metastasis, whereas MHCC97L exhibits 40% of lung metastasis (Li *et al*, 2001). Our data showed that Six1 protein was only expressed in metastatic HCC cell lines (MHCC97L and MHCC97H) but not in nonmetastatic HCC cell lines (Hep3B, Huh7 and PLC). Specific overexpression of Six1 protein in metastatic HCC cells suggested that Six1 may be a metastasis-associated oncogene, which may participate in the process of metastases in HCC. Statistical analysis of the clinicopathological features of HCC patients revealed that overexpression of Six1 protein was significantly correlated with pTNM staging (*P*=0.002) and venous infiltration (*P*=0.004). About 71% of HCC patients recognised at the advanced pTNM stage (45 of 63) or with venous infiltration (42 of 59) were found to overexpress Six1 protein, suggesting that Six1 may play an important role on HCC progression and invasion. Although Six1 protein had not significant association with tumour size (*P*=0.268), more than 60% (48 of 77) of HCC patients with tumours larger than 5 cm showing positive Six1 protein expression (Table 2), indicating its probable function on tumour progression.

Hepatic resection is one of the major curative options for HCC patients. Different centres reported to achieve different survival rates (Llovet *et al*, 2005). Identification of novel risk markers after hepatectomy must be beneficial for management of HCC patients and improvement of their survival. Our result showed that overexpression of Six1 protein in HCC patients was significantly associated with poor overall survival after hepatectomy (*P*=0.0423, Figure 5), suggesting that Six1 may be a novel marker related to poor overall survival of HCC patients after hepatectomy. Although multivariable Cox proportional hazard regression analysis showed that Six1 protein was not an independent factor for predicting the overall survival, logistic regression analysis reflected that Six1 protein was the most influential factor associated with poor 1-year overall survival (*P*=0.013, Table 4) rather than 5-year overall survival (Table 4), proposing that Six1 may be regarded as a predictor for short-term overall survival of HCC patients after hepatectomy. HCC patients with higher rate of early-year death after hepatectomy may be caused by higher malignancy of HCC that pathologically is, in part, determined to be at the advanced pTNM stage or the presence of venous infiltration. Overexpression of Six1 protein in HCC patients was determined to be significantly associated with these malignant factors indicating the possible role of Six1 on promoting HCC malignancy. Even though pTNM staging was determined to be the most important factor in predicting long-term overall survival of HCC patients after hepatectomy (Tables 3 and 4), assessment of Six1 protein level of resected tumour tissues of HCC patients may still provide a valuable index for effective management of HCC patients after hepatectomy especially during early year.

Up to now, understanding of the mechanism of Six1 in pathogenesis of cancers is still limited. Overexpression of Six1 in breast cancer cells can promote the cancer cells to escape from the G2 cell cycle checkpoint after X-ray irradiation (Ford *et al*, 1998). The cell cycle regulatory activity of Six1 in breast cancer is regulated by casein kinase II which inactivates Six1 through phosphorylation (Ford *et al*, 2000). Cyclin A1 is a downstream effector for Six1 in breast cancer where overexpression of Six1 promotes cyclin A1 expression and subsequently increases cell proliferation and progression (Coletta *et al*, 2004). Gene amplification of Six1 is a probable mechanism contributing to tumorigenesis in breast cancer (Reichenberger *et al*, 2005). Overexpression of Six1 in RMS cells can boost their pulmonary metastasis potential, whereas downregulation of Six1 suppresses their metastatic ability (Yu *et al*, 2004). Yu *et al* (2006) also demonstrated that Six1 potentially activates several oncogenes including cyclin D1, c-Myc and Ezrin. Moreover, the ability of Six1 in promoting metastasis of RMS requires the function of Ezrin (Yu *et al*, 2006). The above research evidences in breast cancer and RMS provide important clues to clarify the functional roles of Six1 in HCC because different cancers may have different regulatory mechanisms. In fact, Cyclin A is overexpressed in HCC and its overexpression is associated with poor survival of HCC patients (Chao *et al*, 1998; Ohashi *et al*, 2001). Casein kinase II shows an important involvement of transforming growth factor-beta1-induced HCC (Cavin *et al*, 2003). The relationship between Six1 and Cyclins as well as casein kinase II in regulating the tumorigenesis and metastasis of HCC thus need further clarification. The specificity of Six1 protein expression in HCC metastatic cells may also provide a good bridge to study the functions of Six1 involved in metastasis of HCC through suppression strategy such as using antisense or RNA interference means.

In conclusion, our data indicated that Six1 protein was specifically and frequently expressed in HCC tumour tissues. Overexpression of Six1 protein in HCC patients was significantly associated with advanced pTNM stage and venous infiltration. In addition, Six1 protein could be a novel marker for predicting the short-term overall survival of HCC patients after hepatic resection. Further functional studies are worthwhile to explore the precise mechanism and eventually develop potential therapies targeting at Six1 in liver cancer recurrence and metastases.

## Figures and Tables

**Table 1:** Summary of Six1 expression in liver tissues from HCC patients and normal donors

	**HCC patients**		
**Six1 expression**	**Tumour**	**Nontumour**	**Normal**
*RNA*			
Positive	61 (84.7%)	6 (8.3%)	2 (10 %)
Negative	11 (15.3%)	66 (91.7%)	18 (90%)
			
*Protein*			
Positive	61 (59.2%)	0 (0%)	0 (0 %)
Negative	42 (40.8%)	103 (100%)	20 (100%)

HCC=hepatocellular carcinoma.

**Table 2:** Correlation of Six1 protein expression and clinicopathological features of HCC patients

		**Six1 protein expression (*n*)**		
**Clinicopathological features**	**Number (*n*)**	**Negative**	**Positive**	***P***
*Sex*				
Male	87	35	52	0.792
Female	16	7	9	
				
*Age*				
⩽55 years	59	25	34	0.703
>55 years	44	17	27	
				
*pTNM stage*				
Early stage (I–II)	40	24	16	0.002*
Advanced stage (III–IV)	63	18	45	
				
*Venous infiltration*				
Absent	44	25	19	0.004*
Present	59	17	42	
				
*Cirrhosis* a				
Absent	33	13	20	0.800
Present	69	29	40	
				
*Encapsulation* a				
Absent	48	22	26	0.881
Present	25	11	14	
				
*Tumour size*				
<5 cm	26	13	13	0.268
⩾5 cm	77	29	48	
				
*AFP level*				
⩽20 ng ml^−1^	41	20	21	0.179
>20 ng ml^−1^	62	22	40	
				
*Hepatitis B surface antigen* a				
Negative	16	8	8	0.520
Positive	86	34	52	

AFP=alpha-fetoprotein; HCC=hepatocellular carcinoma; pTNM=pathologic tumour-node-metastasis.

* Significant difference.

a Total number less than 103 due to missing data.

**Table 3:** Cox proportional hazard regression analysis of Six1 protein expression and clinicopathological parameters in relation to the overall survival of HCC patients

	**Univariable analysis**		**Multivariable analysis**	
	**HR (95% CI)**	***P***	**HR (95% CI)**	***P***
*Six1protein*				
Positive *vs* negative	1.956 (1.011–3.784)	0.046	1.29 (0.624–2.503)	NS
				
*pTNM stage*				
Advanced *vs* early	4.952 (2.077–11.808)	0.000	7.698 (1.891–31.33)	0.004
				
*Venous infiltration*				
Presence *vs* absence	3.302 (1.572–6.934)	0.002	0.493 (0.136–1.780)	NS
				
*AFP level*				
>20 ng ml^−1^ *vs* ⩽20 ng ml^−1^	3.062 (1032–4.118)	0.04	1.735 (0.783–3.844)	NS

AFP=alpha-fetoprotein; CI=confidence interval; HCC=hepatocellular carcinoma; HR=hazard ratio; pTNM=pathologic tumour-node-metastasis; NS=not significant.

**Table 4:** Logistic regression analysis of Six1 protein and clinicopathological parameters on predicting the 1- and 5-year overall survival of HCC patients

	**1-year survival**		**5-year survival**	
	**OR (95% CI)**	***P***	**OR (95% CI)**	***P***
*Six1 protein*				
Positive *vs* negative	5.405 (1.427–20.474)	0.013	1.044 (0.401–2.719)	NS
				
*pTNM stage*				
Advanced *vs* early	2.914 (0.245–34.63)	NS	12.152 (1.652–89.378)	0.004
				
*Venous infiltration*				
Presence *vs* absence	1.557 (0.155–15.636)	NS	0.478 (0.072–3.19)	NS
				
*AFP level*				
>20 ng ml^−1^ *vs* ⩽20 ng ml^−1^	1.754 (0.554–5.552)	NS	2.239 (0.846–5.929)	NS

AFP=alpha-fetoprotein; CI=confidence interval; HCC=hepatocellular carcinoma; HR=hazard ratio; pTNM=pathologic tumour-node-metastasis; NS=not significant; OR=odd ratio.
